# Rinse, gel, and foam – is there any evidence for a difference in their effectiveness in preventing infections?

**DOI:** 10.1186/s13756-024-01405-5

**Published:** 2024-05-10

**Authors:** John M. Boyce, Didier Pittet

**Affiliations:** 1 J.M. Boyce Consulting, LLC, Hyde Park, NY USA; 2 Faculty of Medicine, University of Geneva, Geneva, Switzerland

**Keywords:** Alcohol-based hand rub, Rinse, Gel, Foam, Healthcare-associated infections, Effectiveness, Antimicrobial efficacy

## Abstract

**Background:**

Following publication of the 2009 World Health Organizations Guidelines for Hand Hygiene in Health Care, a debate has emerged regarding the relative antimicrobial efficacy of the different formats (rinse, gel, foam) of ABHRs and their ability to contribute to reduction of healthcare-associated infections (HAIs).

**Methods:**

Data regarding the in-vivo antimicrobial efficacy of ABHRs and other factors that likely affect their effectiveness in reducing HAIs were reviewed, and a comprehensive review of studies that reported the effectiveness of each of the three ABHR formats to improve hand hygiene compliance and reduce HAIs was conducted.

**Results:**

The amount of rubbing time it takes for hands to feel dry (dry time) is the major driver of ABHR antimicrobial efficacy. ABHR format is not a major factor, and several studies found that rinse, gel, and foam ABHRs have comparable in-vivo antimicrobial efficacy. Other factors that likely impact the ability of ABHRs to reduce transmission of healthcare-associated pathogens and HAIs include ABHR formulation, the volume applied to hands, aesthetic characteristics, skin tolerance, acceptance by healthcare personnel, and hand hygiene compliance rates. When accompanied by complementary strategies, promoting the use of each of the three ABHR formats has been associated with improvements in hand hygiene compliance rates. A review of 67 studies failed to identify an ABHR format that was significantly more effective in yielding statistically significant reductions in transmission of healthcare-associated pathogens or HAIs.

**Conclusions:**

Current evidence is insufficient to definitively determine if one ABHR format is more effective in reducing transmission of healthcare-associated pathogens and HAIs. More rigorous studies such as multicenter randomized controlled trials comparing the different formats are needed to establish if one format is significantly more effective in reducing HAIs.

## Introduction

Alcohol-based hand rubs (ABHR) have been available for decades in several formats: liquid -also called rinse-, gel, and foam. Despite their availability for years, there is continued debate regarding the effectiveness of gel and foam products in reducing healthcare-associated infections (HAIs). Several publications may have contributed to concerns regarding their effectiveness [1–4]. For example, one early study that compared several gels to four rinses found that none of the ten gels met EN 1500 criteria for efficacy, while all four rinses did [1]. Another early study found that a gel failed EN 1500 efficacy criteria, perhaps due to the low concentration of alcohol (60% isopropanol) [2]. A 2008 controlled trial using a 62% ethanol ABHR gel failed to demonstrate a reduction in device-related infections and multidrug-resistant organism (MDRO) infections [3]. A number of issues have been proposed to explain the lack of apparent efficacy of the gel [5, 6]. A 2013 in vivo laboratory study reported that 1.1 ml of 70% ethanol gel and foam products did not meet U.S. FDA criteria for efficacy, and were inferior to 2 ml of an 85% ethanol ABHR gel [7]. Of note, the methods used were not those required by the FDA, and other studies have found that the 70% gel and foam products meet FDA efficacy standards [8, 9].

The text of the 2009 WHO Guidelines for Hand Hygiene in Health Care notes that rinse, gel and foam ABHRs are available for use in healthcare, and does not recommend one format over another. The 2022 Practice Recommendation: Strategies to Prevent HAIs Through Hand Hygiene published by the Society for Healthcare Epidemiology of America (SHEA), the Infectious Diseases Society of America (IDSA) and the Association for Professionals in Infection Control and Epidemiology (APIC) recommends use of either a liquid, gel, or foam ABHR with at least 60% alcohol [10].

In attempting to answer the question regarding the effectiveness of ABHRs in preventing HAIs, a current literature review failed to identify any controlled trial that compared the effectiveness of different ABHR formats and used the incidence density of HAIs as an outcome measure. In view of the lack of controlled trials to answer the question regarding the relative effectiveness of rinses, gels, and foams to reduce HAIs, the objetive of this paper is two-fold: 1) to discuss factors affecting the in-vivo efficacy and effectiveness of the three ABHR formats in reducing HAIs, and 2) to present a literature review of hand hygiene studies that monitored the frequency of HAIs while using either a liquid, a gel, or a foam ABHR.

## Methods used for literature review

A literature review to identify publications that included information regarding both hand hygiene and HAI rates was conducted by one of the authors (JMB). The PubMed and Google Scholar databases were searched for publications between 2000 and 2023. The following search terms used on the PubMed database included: hand hygiene + healthcare-associated infections; cross infection/prevention & control + hand hygiene + (healthcare-associated infections + rate); cross infection/prevention & control + hand disinfection + (healthcare-associated infections + rate); hand hygiene + healthcare-associated infection rate + prevention; and alcohol-based hand rub + healthcare-associated infections. Google Scholar was searched using the following combination of terms: hand hygiene + healthcare-associated infections + rate. Titles of publications in English were reviewed. Abstracts of articles of potential interest were reviewed, and those determined to be of sufficient interest were downloaded and the text of articles were reviewed. Only studies that reported on HAI rates and information regarding hand hygiene compliance were noted. Bibliographies of downloaded articles were also reviewed for pertinent studies. Authors of 45 publications of interest which did not provide data on the ABHR format used were sent emails requesting information regarding the ABHR format utilized during the respective study periods.

Data recorded for each study included the publication year, first author, ABHR format used, study time period(s), the country or countries in which the study occurred, the impact on hand hygiene compliance rates and on various healthcare outcomes. The impact on MDRO colonization/acquisition rates was included as a healthcare outcome, because such rates are directly affected by the effects that hand hygiene has on transmission of pathogens. Differences in proportions were analyzed using Chi-square tests, while continuous data were compared using the Kruskal–Wallis test.

## Results

### Factors affecting antimicrobial efficacy

Factors that have been shown to affect the ability of ABHRs to reduce bacterial counts on hands include the volume of ABHR applied to hands, the amount of time that hands must be rubbed together before they feel dry (so-called dry time), ABHR formulation, and methods used to conduct in-vivo efficacy studies [11, 12].

#### Volume of ABHR applied

Multiple studies have demonstrated that the greater the volume of ABHR applied to hands, the greater the log_10_ reduction of viable bacteria on hands [13–19]. Increasing the volume of ABHR applied to hands also increases dry times [16–21].

#### Dry time

The antimicrobial efficacy of ABHRs is also affected by dry times [13, 16, 18, 20]. Importantly, dry time is a major driver of antimicrobial efficacy, independent of the volume applied [18]. To achieve acceptable antimicrobial efficacy, the WHO Guideline for Hand Hygiene in Health Care recommends that dry times should be a minimum of 20 s when performing hand hygiene with an ABHR [22], while the 2022 SHEA/IDSA/APIC Practice Recommendation on hand hygiene recommends that dry times should be a minimum of 15 s [10]. Since some healthcare personnel rub their hands together for less than 5 to 10 s [23–25], hand hygiene education programs should emphasize the importance of achieving recommended dry times.

#### ABHR formulation

Several studies have found that ABHR formulation can influence antimicrobial efficacy [8, 16, 26]. For example, Edmonds et al. [8] found that a novel 70% ethanol gel yielded significantly higher log_10_ reductions after one and after 10 applications than two other 70% ethanol gel formulations, and that a novel 70% ethanol foam yielded significantly higher log_10_ reductions after one and 10 applications than another 70% ethanol foam formulation. Barbut et al. [26] found that a rinse formulation containing 30% n-propanol, 45% isopropanol and 0.2% mecetronium ethyl-sulphate (total alcohol content 75%) and a gel formulation containing a higher alcohol content (85% ethanol) yielded nearly identical log_10_ reductions, despite the higher total alcohol content of the latter. Macinga et al. [27] found that gel formulations ranging from 62%, 70% and 85% ethanol yielded log_10_ reductions that were not significantly different. Another study revealed that a 70% ethanol foam yielded a mean log_10_ reduction that was not statistically different from those achieved by an 80% ethanol rinse, and a 90% gel, demonstrating that formulation constituents other than alcohol can affect efficacy [16]. Overall, the above studies demonstrate that formulation is more important than alcohol concentration alone in determining antimicrobial efficacy.

ABHR formulation can also affect dry times [16, 28, 29]. Dry times vary when different ABHR products are applied with the same volume [28]. ABHRs with higher ethanol concentrations dry faster, regardless of the format [16].

#### Does ABHR format (rinse, gel, foam) affect efficacy?

A 2002 study using the EN 1500 method compared the log_10_ reductions achieved by 10 “first generation” gels with alcohol concentrations from 53 to 70% and by four rinses containing from 64 to 75% alcohol to the reference alcohol (60% 2-propanol). None of the gels met EN 1500 criteria for efficacy, while all four rinses did [1]. Based on the study results, efforts were made to improve the antimicrobial efficacy of gels. Of note, foams were rarely used when the study was performed.

Several more recent studies have shown that ABHR format (rinse, gel, foam) does not have a major impact on in vivo antimicrobial efficacy [8, 16, 29, 30]. One study that evaluated the effect of different ASTM test methods on antimicrobial efficacy of a gel and a foam (both 70% ethanol) found that the test method used significantly affected antimicrobial efficacy, but ABHR format did not [30]. Table 1 shows the results for three studies that assessed the influence of product format on antimicrobial efficacy by using the EN 1500 method [8, 16, 29].

**Table 1:** Log_10_ reductions achieved by liquid, gel and foam ABHRs in three studies using EN 1500 methods

Author	Format	Application Volume (ml)	Mean log_10_ Reduction at 3 ml	Mean log_10_ Reduction at 3 ml × 2	*P* value
Edmonds [8]	Gel – 70% ETOH	3	5.25	5.11	N.S.D
	Gel – 70% ETOH	3	5.17	4.8	N.S.D
	Foam – 70% ETOH	3	5.06	5.11	N.S.D
Macinga [16]	Rinse – 60% IPA	3 ml × 2		4.63	
	Rinse – 80% ETOH	3	4.50		0.68*
	Gel – 90% ETOH	3	4.61		0.99*
	Foam – 70% ETOH	3	4.56		0.93*
Wilkinson [29]	Rinse – 60% IPA	3	4.19		N.S.D
	Gel – 60% IPA	3	4.26		N.S.D
	Foam – 60% IPA	3	4.22		N.S.D
	Rinse – 80% ETOH	3	4.03		N.S.D
	Gel – 80% ETOH	3	4.52		N.S.D
	Foam – 80% ETOH	3	4.34		N.S.D

*ETOH* Ethanol, *IPA* Isopropanol, *N.S.D.* Not statistically different

^*^P value compared to EN 1500 reference solution

Accordingly, if ABHR format makes a difference in the abilities of ABHR rinses, gels, and foams to reduce HAIs, it is unlikely to be due to differences in antimicrobial efficacy. Several studies suggest that ABHR format is not a major determinant of dry time [16, 26, 31]. The above study by Wilkinson et al. [29] found rinses or foams were perceived by HCP to dry more quickly than gels, but format did not result in significant differences in antimicrobial efficacy. 

#### Methods for in-vivo efficacy studies

The in-vivo methods used to study ABHRs can also affect conclusions regarding their efficacy [8, 18, 27, 30]. For example, using a smaller volume of fluid to artificially contaminate hands can yield greater log_10_ reductions [27]. Log_10_ reductions achieved with the ASTM E1174 method can differ substantially compared with those obtained with the EN 1500 standard [8]. And ASTM methods E1174, E2755 and E2784 yield different estimates of efficacy [30]. In addition, a modified EN 1500 protocol based on rubbing hands until dry found that log_10_ reductions were greater when larger volumes of ABHR were applied [18].

### Factors that likely impact ABHR effectiveness

In addition to antimicrobial efficacy, a number of other factors are likely to influence the ability of ABHRs to reduce HAIs (i.e., their effectiveness) [11, 12].

#### Level of acceptance by healthcare personnel

Poor skin tolerance (dryness or irritation), an unpleasant feeling of ABHR during or after application, an unfavorable smell and prolonged drying time, all of which can be affected by ABHR formulation, can affect healthcare personnel (HCP) preferences and may reduce acceptance by HCP [26, 32–38]. HCP prefer products that are least likely to result in skin dryness compared to those that are prone to cause dryness with repeated use [32, 39]. Some HCP prefer ethanol-based ABHRs to isopropanol-containing agents based on differences in their smell [26, 37]. HCP prefer ABHRs that are easier to contain in the hand and less likely to drip [36, 37]. HCP are less likely to use a product with a lower acceptance rate [26]. Higher levels of acceptance of an ABHR can lead to greater hand hygiene compliance [34].

#### Volume of ABHR delivered

The volume of ABHR applied, an important factor in achieving the desired antimicrobial efficacy, can vary substantially based on the amount HCP choose to use and by the volume delivered by dispensers [9, 40–45]. Application of suboptimal volumes of ABHR by HCP will decrease the antimicrobial efficacy of hand hygiene [15, 18], and may adversely affect the impact of hand hygiene on prevention of HAIs.

#### Hand hygiene compliance rates by HCP

Multiple studies have found that the rate of hand hygiene compliance by HCP can influence transmission of healthcare-associated pathogens and HAIs, with reductions in transmission and HAIs occurring with higher compliance rates [46–48].

A combination of the above factors likely impacts the ability of ABHRs, independent of their antimicrobial efficacy or format, to reduce HAIs. In addition, there is substantial evidence that most facilities that have improved hand hygiene compliance rates and reduced transmission of healthcare pathogens and/or HAIs have implemented multimodal hand hygiene improvement strategies such as the one recommended by the WHO Guidelines for Hand Hygiene in Health Care [22], and have often implemented other strategies not related to hand hygiene [49]. As a result, the availability of ABHR, accompanied by little or no additional complementary components, is not likely to improve hand hygiene compliance [50], and will probably have little impact on HAIs.

## Results of the literature review

Database searches yielded a combined total of 1,948 titles, with review of 318 abstracts. The full texts of 114 articles were downloaded. Emails requesting data on ABHR format were sent to 45 authors, of whom 34 responded. Data from 67 publications are included in the review. The primary results of the literature review are presented in Table 2.

**Table 2:** Publication year, ABHR format, study time period, country in which the study was performed, impact on hand hygiene compliance and healthcare outcome

**Pub Date**	**First Author**	**ABHR Format**	**Time period**	**Country**	**Healthcare Outcome**
2000	Pittet [46]	liquid	1993–1998	Switzerland	Significant decrease in HAIs and MRSA transmission
2004	Won [51]	liquid	1997–2001	Taiwan	Significant decrease in HAIs
2007	Pessoa-Silva [52]	liquid	Mar 2001-Feb 2004	Switzerland	Decrease in BSIs in NICU
2008	Nguyen [53]	liquid	Apr—Dec 2007	Vietnam	Significant decrease in HAIs
2009	Souweine [54]	liquid	Apr 2003-Jan 2004	France	Decrease in MRSA colonization/infection
2010	Cheng [55]	liquid	Jan 2002-Jun 2009	Hong Kong	Significant decrease in MRSA infections, including MRSA BSIs
2011	Chen [56]	liquid	Jan 1999-Dec 2007	Taiwan	Significant decrease in HAIs
2011	Barrera [57]	liquid	Feb-Jun 2002	Columbia	Significant decrease in CLABSIs, not other HAIs
2012	Monistrol [58]	liquid	Feb 2007-Jan 2008	Spain	Decrease in MRSA acquisition; no change in HAIs
2012	Ho [59]^a^	liquid	Nov 2009-Jul 2010	Hong Kong	Significant decrease in respiratory and MRSA infections
2012	Ling [60]	liquid	Jan 2007-Dec 2010	Singapore	Decrease (borderline) in HA-MRSA infections
2012	Lee [61]	liquid	Jan 2004-Dec 2010	Taiwan	Significant decrease in HA-MRSA infections
2015	Thi Anh Thu [62]	liquid	Jun 2009-Apr 2011	Vietnam	Significant decrease in HAIs
2015	Sadeghi-Moghaddam [63]	liquid	Apr 2010-Mar 2014	Iran	Significant decrease in HAIs
2016	Cheng [64]	liquid	Jul 2011-Oct 2015	Hong Kong	Significant decrease in VRE transmission
2017	Saito [65]	liquid	Oct 2014-Apr 2015	Uganda	Significant decrease in HAIs/SIRs in pediatric & surgical dept
2018	Allegranzi [66]	liquid	Jul 2013-Dec 2015	African countries	Significant decrease in surgical site infections
2018	Grayson [67]	liquid	2009–2017	Australia	Significant decrease in healthcare-associated *S. aureus* bacteremia
2019	von Lengerke [68]^a^	liquid	2013–2015	Germany	Significant decrease in MDROs
2019	Hagel [69]	liquid	Sep 2011-Aug 2012 May 2013-Aug 2014	Germany	No change in overall HAIs; Significant decrease in severe ICU HAIs
2020	Aghdassi [70]^a^	liquid	Dec 2017-Dec 2018	Germany	Significant decrease in device-related BSIs
2020	Phan [71]	liquid	2010–2018	Vietnam	Significant decrease in HAIs
2021	Knudsen [72]	liquid	Feb 2020-Jan 2021	Denmark	Significant decrease in HAIs
2021	Lo [73]	liquid	2018-May 2020	Taiwan	Significant decrease in CRAB and VRE infections
2022	Fukushige [74]	liquid	2018–2020	Taiwan	Significant decrease in overall HAIs; not in MRSA or VRE HAIs
2022	Gonzalez-Gonzalez [75]	liquid	2009–2019	Mexico	Significant decrease in CLABSIs
2023	Rosenfeldt Knudsen [76]	liquid	Feb 2020-Jun 2022	Denmark	Significant decrease in HABSIs; no significant change in CAUTIs
2002	Fendler [77]	gel	Jul 1997-May 2000	USA	Significant decrease in HAIs
2003	Hilburn [78]	gel	Feb 2000-May2001	USA	Significant decrease in HAIs
2004	MacDonald [79]	gel	Mar 2000-Nov 2000	Scotland	Significant decrease in MRSA acquisition
2007	Murthy [80]	gel	1999–2003	USA	Significant decrease in nosocomial MRSA BSIs
2007	Harrington [81]	gel	Jan 2003-May 2007	Australia	Significant decrease in MRSA BSI
2008	Rupp [3]	gel	Aug 2001-Sep 2003	USA	No change in device-related infections/MDROs/CDI
2009	Al-Naami [82]	gel	Oct 2007-Juh 2008	Saudi Arabia	Decrease in surgical site infections
2010	Carboneau [83]	gel	Mar 2006-Feb 2007	USA	Non-significant decrease in nosocomial MRSA infections
2011	Yeung [84]^a^	gel	Jan 2007 -Oct 2007	Hong Kong	Significant decrease in HAIs
2011	Marra [85]	gel	Jul 2008-Dec 2009	Brazil	Significant decrease in device-related HAIs
2011	Garcia–Vazquez [86]	gel	Not stated	Spain	Significant decrease in HAIs
2012	Kirkland [87]	gel	Jan 2006-Nov 2009	USA	Significant decrease in HAIs
2012	Mestre [88]	gel	Mar 2007-Dec 2011	Spain	Significant decrease in healthcare-acquired MRSA
2013	Talbot [89]	gel	Jan 2007-Aug 2012	USA	Significant decrease in device-related infections
2013	Salama [90]	gel	Feb 2011 – Aug 2011	Kuwait	Significant decrease in HAIs
2013	Iacobelli [91]	gel	Jan 2007-Dec 2009	France	Significant decrease in MRSA infections and colonization
2014	Johnson [92]	gel	Apr 2006—Sep 2012	USA	Significant decrease in CLABSIs
2015	Fox [93]	gel	Dec 2009-Feb 2012	USA	Decrease in CLABSIs and CAUTIs
2015	Chun [94]	gel	Oct 2008-Jan 2009	South Korea	Significant decrease in MRSA acquisition and colonization pressure
2016	Shabot [95]	gel	Oct 2010-Dec 2014	USA	Significant decrease in ICU CLABSI and VAP
2018	Ragusa [96]	gel	2015–2016	Italy	Increase in CDI
2018	Al-Tawfiq [97]	gel	2014-Sep 2015	Saudi Arabia	Decreases in CDI, CLABSIs and CAUTIs
2018	De la Rosa-Zamboni [98]	gel	Jan 2013—Aug 2013	Mexico	Significant decrease in HAIs
2018	Lenz [99]	gel	Jun 2011-Apr 2012	Argentina	Significant decrease in CABSIs
2020	Ojanpera [100]	gel	2013–2018	Finland	Significant decrease in HAIs
2021	Banks [101]	gel	Jun 2017-Jul 2019	USA	Significant decrease in CDI
2021	Akkoc [102]	gel	Apr 2016—Aug 2016	Turkey	Significant decrease in HAIs, with non-significant decrease in device-related infections
2023	Ragonese [103]	gel	Nov 2017-Dec 2020	Italy	Significant decrease in HA-CRE colonization rate
2004	Swoboda [104]	foam	Jul 2000-Oct 2001	USA	Significant decrease in MRSA or VRE infection/colonization; non-significant decrease in overall HAIs
2010	Helms [105]	foam + gel	Aug 2007-Jun 2008	USA	Non-significant decrease in HAIs
2010	Knight [106]	foam	Jan 2001-Jun 2008	USA	Significant decrease in HO-CDI
2013	Raschka [107]	gel—> foam	2007–2011	Canada	Non-significant decrease in selected HAIs
2013	Schweon [108]	foam	May 2009-Feb 2011	USA	Significant decrease in lower respiratory infections; Decrease in skin & soft tissue infections
2016	Sickbert-Bennett [109]	foam	Oct 2013-Feb 2015	USA	Significant decrease in HAIs
2016	Kelly [110]	foam	Jul 2012-Mar 2015	USA	Significant decrease in MRSA infections
2018	McCalla [111]	foam	Jan 2014-Sep 2017	USA	Significant decrease in CLABSIs and CAUTIs
2019	Boyce [112]	foam	Jun 2014-Jun 2018	USA	Decrease in non-CDI; increase in CDI
2020	Leis [113]^a^	foam	Jun 2017-Dec 2018	Canada	Non-significant decrease in MRSA colonization/infection; not HA-BSIs or CDI
2020	Knepper [114]	foam	July 2016-Dec 2017	USA	Significant decrease in CDI. No change in other HAIs
2023	Barrett [115]	foam	Feb 2018-Sep 2021	USA	Significant decrease in MRSA infections

^a^Healthcare Outcome refers to reduced transmission of healthcare pathogens and/or healthcare-associated infections. *HAIs* Healthcare-associated infections, *MRSA* Methicillin-resistant *S. aureus*, *BSIs* Bloodstream infections, *NICU* Neonatal intensive care unit, *CLABSIs* Central line-associated bloodstream infections, *HA-MRSA* Healthcare-associated MRSA, *VRE* Vancomycin-resistant enterococci, *SIRs* Standardized infection ratios, *MDROs* Multidrug-resistant organisms, *ICU* Intensive care unit, *CRAB* Carbapenem-resistant *Acinetobacter baumannii*, *HABSIs* Healthcare-associated BSIs, *CAUTIs* Catheter-associated urinary tract infections, *CDI Clostridioides difficile* infection, *VAP* Ventilator-associated pneumonia, *CABSIs* Catheter-associated BSIs, *HA-CRE* Healthcare-associated carbapenem-resistant *Enterobacteriaceae*, *HO-CDI* Hospital-onset CDI

The ABHR formats used in the 67 studies were rinses (*n* = 27), gels (*n* = 28) and foams (*n* = 12). The proportion of studies published > 10 years ago was slightly, but not significantly, higher for rinses (44.4%) and gels (57.1%) than for foams (41.7%) (*p* > 0.05).

The duration of the study periods ranged from 4 months to 11 years, with the following median study durations (in months): rinses = 43.2; gels = 28.2; foam = 34.3 (*p* > 0.05). The countries in which the studies were conducted differed significantly for the different formats. None of 27 studies using rinses were conducted in the United States or Canada, whereas 11/28 (39.3%) of gel studies and 12/12 (100%) of foam studies were conducted in North America (*p* < 0.001). Hand hygiene compliance rates were not reported in 14 studies, although 9 of these reported increased ABHR consumption. Of the remaining 53 studies, 51 reported improved hand hygiene compliance rates, one study utilizing a rinse reported no significant change, and one utilizing a gel reported a decrease in compliance.

Of the 67 studies, all but two gel studies, one of which found no improvement in hand hygiene compliance, reported some decrease in HAIs or MDRO colonization/acquisition rates (Table 2). However, not all studies that reported decreases reported a statistically significant decrease in healthcare outcomes. The proportion of studies in which a significant decrease in one or more healthcare outcomes occurred was as follows: rinses (23/27, 85.2%); gels (22/28, 78.6%); and foams (8/12; 75%) (*p* > 0.05).

## Discussion

In the intervening years since the WHO Guidelines for Hand Hygiene for Health Care was published in 2009, a debate has emerged regarding the relative antimicrobial efficacy of the different formats of ABHRs and their ability to contribute to reduction of HAIs. Recent evidence suggests that ABHR dry time is the primary driver of antimicrobial efficacy, independent of the volume applied [18]. Furthermore, additional studies provide evidence that ABHR rinses, gels, and foams have comparable in-vivo antimicrobial efficacy [8, 16, 29, 30]. Accordingly, it may be reasonable to assume that any potential differences in the ability of the different ABHR formats to contribute to the reduction of healthcare-associated pathogen transmission and HAIs are unlikely to be related to their antimicrobial efficacy, provided that those ABHRs meet the ASM or EN norms. Other factors more likely to affect the ability of different ABHRs to contribute to HAI reduction include the volume of handrub applied to the hands, dry times, product formulation, acceptance by HCP, and resulting hand hygiene compliance rates. HCP prefer ABHRs that dry quickly, so that hand hygiene action takes less time. But currently available data are somewhat contradictory regarding which format routinely provides the shortest dry times. For example, one study found similar dry times for the 3 formats [16], while others have reported that dry times appeared to be shorter for rinses [38], or that rinses and foams dry more quickly than gels [29].

The striking differences in the geographical locations in which the studies of the different formats were conducted (no rinse studies and all foam studies conducted in North America) is an interesting finding, the explanation for which was not provided by the review. Factors that may help explain this phenomenon include efficacy results obtained with different testing protocols (EN1500 in Europe vs ASTM methods often used in the United States), cultural considerations, influence of local and regional hand hygiene experts, and marketing strategies of ABHR manufacturers. The review suggests that the antimicrobial efficacy of the three formats is unlikely to explain this phenomenon.

The literature review has several limitations. Not all published studies reporting the impact of hand hygiene on HAI rates were included due to the lack of available data regarding the ABHR format used. Compared to rinses and gels, there were substantially fewer studies that involved the use of ABHR foams, which may have affected the results. There is considerable heterogeneity in the study designs utilized and in the completeness of data provided. Of the 67 studies, only 4 were cluster randomized controlled trials [59, 68, 70, 113], and one was a prospective, controlled trial with cross-over design [3]. Most of the studies were before-after quasi-experimental observational studies. No statistical analysis was provided in several studies (rinse – 1, gel – 1, foam – 2) [60, 83, 105, 107]. precluding the ability to determine if decreases in HAIs were statistically significant. A majority of the studies did not report the alcohol concentration of the ABHRs used. However, 28 studies (performed between 1993 and 2022) that reported product details used ABHRs with alcohol concentrations ranging from 60 to 95% (most were 62% to 75%), which is within the range that the WHO hand hygiene guideline regards as the most effective [22]. In addition to providing ABHRs available to HCP, virtually all studies implemented additional complementary strategies such as those recommended by the WHO [22, 116], making it impossible to attribute decreases in healthcare outcomes solely to the availability, antimicrobial efficacy, or format of ABHR. 

Due to its multiple shortcomings, the literature review failed to identify a format that was significantly better in yielding statistically significant reductions in the healthcare outcomes measured. The limitations of the literature review make it clear that much more rigorous methods are needed to identify which format reduces HAI most effectively, if in fact any differences exist.

To definitively answer if any format is significantly more effective, a prospective multicenter, cluster randomized controlled trial of 6 to 12 months duration would most likely be needed to determine if ABHR format, by itself, is an independent predictor of effectiveness. Importantly, in healthcare institutions where HAI rates are extremely low, the study duration might be even longer, ie. up to 36 months. If it were feasible, including a cross-over feature and performance in different countries would be desirable. Such a trial would need to address multiple potential confounding factors. Ideally, study sites would need to have comparable patient populations, hand hygiene compliance rates obtained with similar methods, HAI definitions and surveillance methods. And the ABHR rinses, gels and foams tested should have comparable in-vivo antimicrobial efficacy, dispensing systems delivering the same volumes of product, and HCP acceptance rates. The likelihood that such a trial will be conducted appears to be slim at best.

As pointed out recently, there are several other issues related to ABHRs that warrant further research that should be feasible [117]. There is a need for additional high-quality evidence-based trials to compare the in-vivo antimicrobial activity of rinses, gels, foams and sprays, and new clinical studies to assess the skin tolerance and HCP acceptance of ABHRs, their frequency of use, and the impact of these factors on hand hygiene compliance rates [117]. Because there is increasing evidence that ABHR dispensers which deliver the same dose to HCP may result in suboptimal dry times in HCP with relatively large hands, continued efforts to develop dispensers capable of dispensing volumes based on an individual healthcare worker’s hand size are needed [18, 117].

## Conclusions

ABHR format is not a major driver of antimicrobial efficacy, provided the product meets recommended standards [22, 118, 119]. In light of studies suggesting that properly formulated rinse, gel, and foam ABHRs have similar antimicrobial efficacy, it is perhaps not surprising that all formats have been associated with successful efforts to improve hand hygiene compliance, when incorporated into a multimodal improvement strategy. Current evidence is insufficient to definitively determine if one ABHR format is more effective in reducing transmission of healthcare-associated pathogens and HAIs.

## Data Availability

No datasets were generated or analysed during the current study.
